# Βeta 2-glycoprotein I protects mice against gram-negative septicaemia in a sexually dimorphic manner

**DOI:** 10.1038/s41598-017-07945-8

**Published:** 2017-08-15

**Authors:** Fatima El-Assaad, Miao Qi, Alice Kizny Gordon, Jian Qi, Shangwen Dong, Freda Passam, James Crofton Weaver, Bill Giannakopoulos, Steven Anthony Krilis

**Affiliations:** 1 St George and Sutherland Clinical School, Faculty of Medicine, University of New South Wales Australia, Research and Education Centre, Sydney, New South Wales Australia; 2 Department of Infectious Disease, Immunology and Sexual Health, St George Hospital, Kogarah, New South Wales Australia; 3 Department of Microbiology, South Eastern Area Laboratory Service, St George Hospital, Kogarah, New South Wales Australia; 4 Department of Cardiology, St. George Hospital, Kogarah, New South Wales Australia; 5 Department of Rheumatology, St. George Hospital, Kogarah, New South Wales Australia; 6 Department of Cardiothoracic Surgery, Tianjin Medical University General Hospital, Tianjin, China

## Abstract

The immune responses of males and females to bacterial infections display differences. The mechanisms that underlie this sexual dimorphism are multifactorial. Lipopolysaccharide (LPS) contributes to the pathogenesis of endotoxaemia. We have previously demonstrated that the plasma protein beta-2 glycoprotein-1 (β2GPI) reduces LPS-induced inflammation in male mice. In the present study using a more robust infection model of septicaemia the role of β2GPI is examined in both male and female wild type (WT) and β2GPI deficient (β2GPI^−/−^) mice challenged with *Escherichia coli* (*E. coli*) intravenously. β2GPI deficiency led to an increase of *E. coli* colony forming units (CFU) in the circulation of both male and female mice. In male β2GPI^−/−^ mice this was associated with a worse clinical severity score. This difference was not observed between female β2GPI^−/−^ and female WT mice. Male WT mice had decreased levels of total and increased levels of free thiol β2GPI following administration of LPS or *E. coli*. This pattern of sexual dimorphic response was also observed in our cohort of humans with sepsis. These findings support a role for β2GPI in modulating the sex-specific susceptibility to gram-negative septicaemia.

## Introduction

Gram-negative bacterial infection in the blood (septicaemia) leading to sepsis is an underestimated health risk and the main cause of death in hospital intensive care units (ICU) worldwide^1^. Sepsis accounts for an estimated 5.3 million deaths per year and surviving patients suffer long-term complications^1^. Sepsis is an aggressive dysregulated systemic inflammatory response that is characterized by fevers, chills, confusion, difficulty breathing, altered mental state, systemic hypotension and multiple organ failure^2^.

Recently, two independent groups discovered that beta 2-glycoprotein 1 (β2GPI), an abundant plasma protein, could interact with and attenuate immune responses to lipopolysaccharide (LPS) in humans^3^ and mice^4^.

β2GPI, also known as apolipoprotein H (43 kDa) is produced by the liver, circulates in the blood (200 μg/mL) and is highly conserved across species^5–8^. The LPS binding site sequence in β2GPI is identical in all mammals^3^. β2GPI consists of five domains (domain I at the N-terminus through to domain V at the C-terminus) that are complement control protein repeats. It exists in two functionally distinct forms; free thiol and oxidized β2GPI^9, 10^. Free thiol β2GPI is the major form circulating in plasma and has been shown to protect human umbilical vein endothelial cells and retinal epithelial cells from hydrogen peroxide induced death^10, 11^. The fifth domain of β2GPI has an extra disulphide bond between Cys288 - Cys326 at the C terminus, which has been demonstrated to be reduced by the oxidoreductases thioredoxin (TRX-1) and protein disulphide isomerase (PDI)^9, 12^. Male mice given an intravenous injection of LPS have increased plasma levels of free thiol β2GPI^4^.

There are sexually dimorphic immunological responses to a variety of infections that have recently been reviewed^13^.

This study is the first to examine the influence of β2GPI in a murine model of gram-negative septicaemia using live *E. coli* organisms rather than LPS alone. Male and female wild type (WT) and β2GPI deficient (β2GPI^−/−^) mice were challenged with *E. coli* and monitored for up to 24 h. We demonstrate a sexually dimorphic role for β2GPI in murine gram-negative septicaemia and present corroborative findings in human sepsis.

## Results

### β2GPI deficiency predisposes to an early onset of severe gram-negative septicaemia in male mice

**Figure 1:**
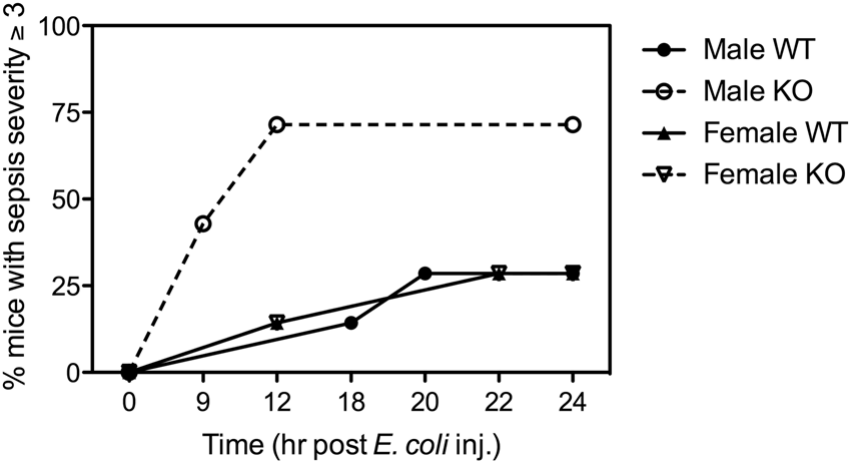
β2GPI confers protection against gram-negative bacterial septicaemia in male mice. Septicaemia clinical severity time course over 24 h of male and female WT and β2GPI^−/−^ mice following intravenous administration with 10^8^ CFU of *E. coli*. Mice were euthanized when they reached a severity score of ≥3. A greater percentage of male β2GPI^−/−^ developed severe septicaemia compared to male WT mice following *E. coli* injection (*n = *7 per group, *p = *0.02, Gehan-Breslow-Wilcoxon Test). There was no difference in septicaemia clinical severity score between female WT and female β2GPI^−/−^ mice. Male WT = ●, Male β2GPI^−/−^ = ○, Female WT = ▲, Female β2GPI^−/−^ = ▽.

Male and female WT and β2GPI^−/−^ mice were monitored and assigned a sepsis severity score over 24 h following an intravenous injection of *E. coli* (Table 1). Mice were sacrificed when they reached a severity score of ≥3. Male β2GPI^−/−^ mice developed severe septicaemia earlier than male WT mice within 24 h following an intravenous injection of *E. coli* (at 24 h male β2GPI^−/−^ 71.4% *vs* male WT 28.6%, had a severity score of ≥ 3, p < 0.03, *n = *7) (Fig. 1). In contrast, there was no difference in severity score in female β2GPI^−/−^ compared to female WT mice (female β2GPI^−/−^ 28.6% *vs* female WT 28.6%, *p = ns*, *n = *7 per group) (Fig. 1).

**Table 1:** Sepsis severity score.

Score	Observation
0	No discernible clinical signs Smooth coat Active mouse ormal stimulus response Open eyes Normal respiration Normal posture
1	Slightly ruffled coat (piloerection) Slightly slowed activity Strong response to touch (moves to escape) Open eyes Normal respiration Normal posture
2	Ruffled coat Slowed activity Mild response to touch (moves only a few steps) Eyes half closed Slight decrease in respiration Hunched posture
3	Ruffled coat Mouse is stationary upright, isolated from group Little or no response to touch (abnormal gait) Eyes half or fully closed, secretions may be present Laboured respiration Hunched posture
4	Ruffled coat may not be present, emancipated Mouse is stationary, isolated from group No response to touch, cold to the touch Eyes closed, secretions may be present Laboured respiration with long gaps May not be upright

### The role of β2GPI in the clearance of E. coli is sexually dimorphic

**Figure 2:**
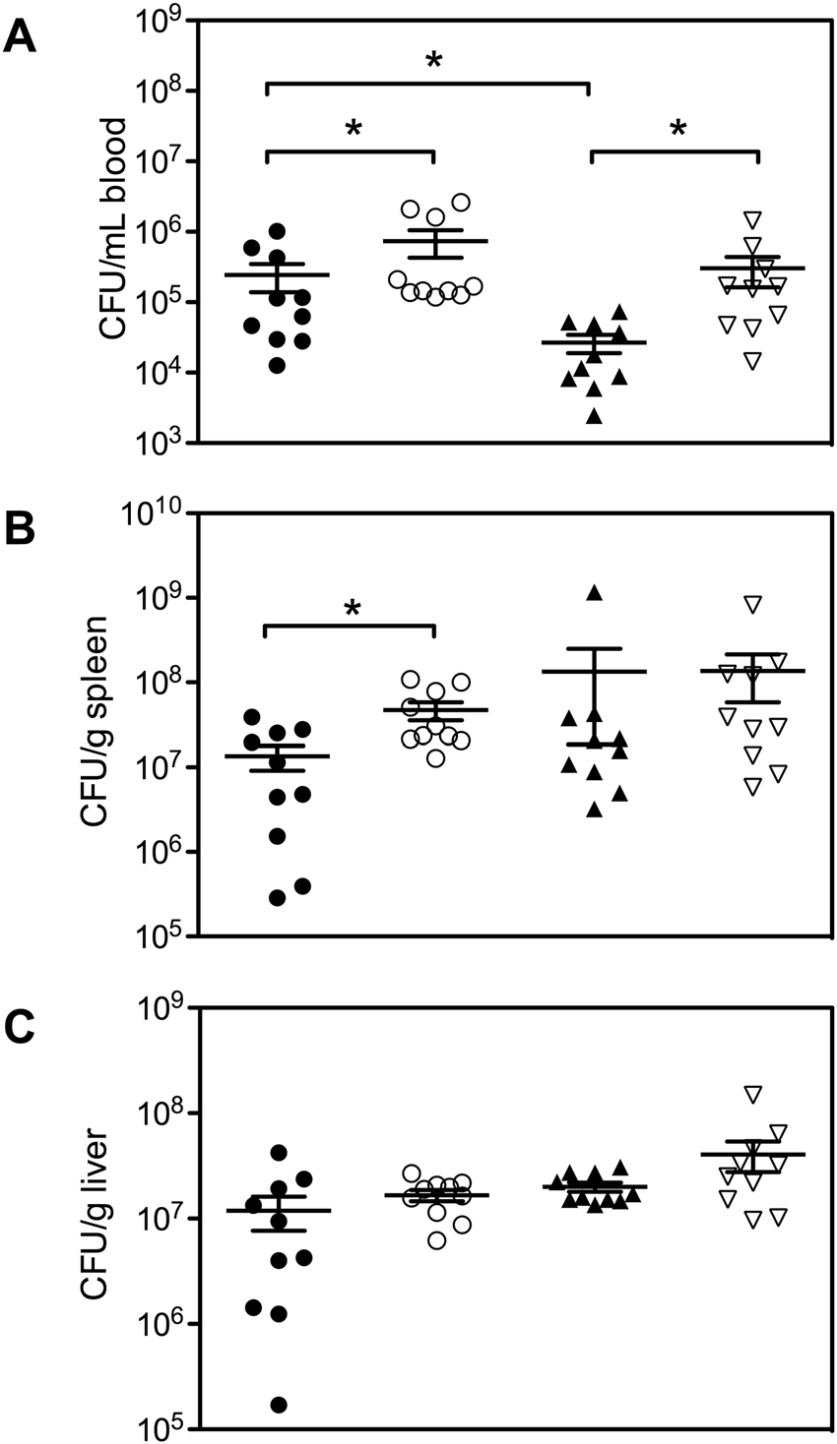
The effect of β2GPI deficiency in the clearance of *E. coli*. Mice were infected with 10^8^ CFU of *E. coli* or an equivalent volume of pyrogen-free saline as controls. Blood, spleen and liver were collected aseptically six h post infection, homogenized and cultured on HBA plates overnight. CFU were determined blind and normalized to blood volume or spleen/liver weight. (**A**) Clearance of *E. coli* from the blood. (**B**) Clearance of *E. coli* CFU in the spleen (**C**). Clearance of *E. coli* CFU in the liver. Data was log transformed and is represented as mean and SEM (*n = *10 per group; Mann-Whitney *p < 0.05). Male WT = ●, Male β2GPI^−/−^ = ○, Female WT = ▲, Female β2GPI^−/−^ = ▽.

We found that female WT mice cleared *E. coli* more efficiently from the peripheral circulation than male WT mice (female WT 26, 647 ± 7, 762 CFU/mL *vs* male WT 244, 906 ± 105, 814 CFU/mL, mean ± SEM, *n = *10, *p < *0.01) (Fig. 2A). This difference was not observed in female β2GPI^−/−^ compared to male β2GPI^−/−^ mice (Fig. 2A). Splenic homogenates from β2GPI^−/−^ male mice produced more bacterial CFU than splenic homogenates from male WT mice (male β2GPI^−/−^ 4.7 × 10^7^ ± 1.1 × 10^7^ CFU/g *vs* male WT 1.3 × 10^7^ *± *4.3 × 10^6^ CFU/g mean ± SEM, *n = *10, p < 0.05) (Fig. 2B). We found no difference in the number of bacterial CFU produced from splenic homogenates between female WT and female β2GPI^−/−^ mice or liver homogenates across all groups (Fig. 2B,C).

### β2GPI has a sexually dimorphic influence on the spleen weight of mice following an E. coli challenge

**Figure 3:**
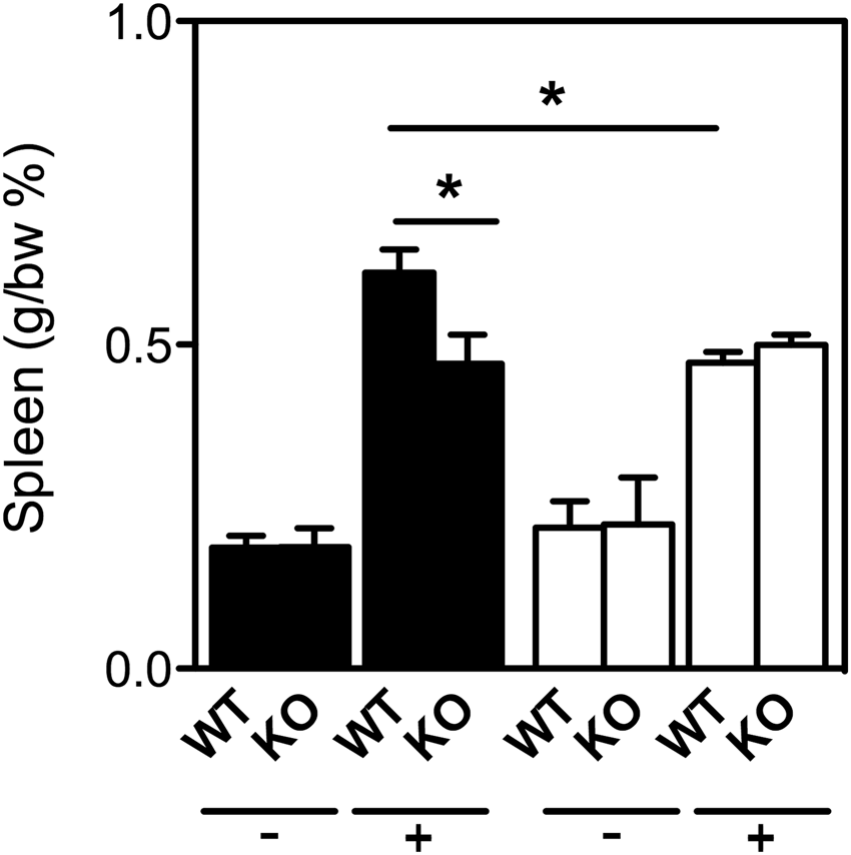
β2GPI has a sexually dimorphic influence on the spleen weight of mice following an *E. coli* challenge. Spleen weight (gram weight as a percentage of body weight (g/bw)) of WT and Β2GPI^−/−^ mice six h after *E. coli* intravenous injection (*n* = 10 per group). Male = ▬, Females = ▭, “−”  = saline, “ + ” = *E. coli*. Data represented as mean ± SEM, 1 way ANOVA, Tukey’s Multiple Comparison Test; *p < 0.05.

Spleen weight of male WT mice was higher than male β2GPI^−/−^ mice following an *E. coli* challenge (*n = *10, p < 0.05). No difference was observed between female WT and female β2GPI^−/−^ mice (*n = *10, p > 0.05) (Fig. 3).

### Plasma levels of total and free thiol β2GPI in murine septicaemia and human sepsis

**Figure 4:**
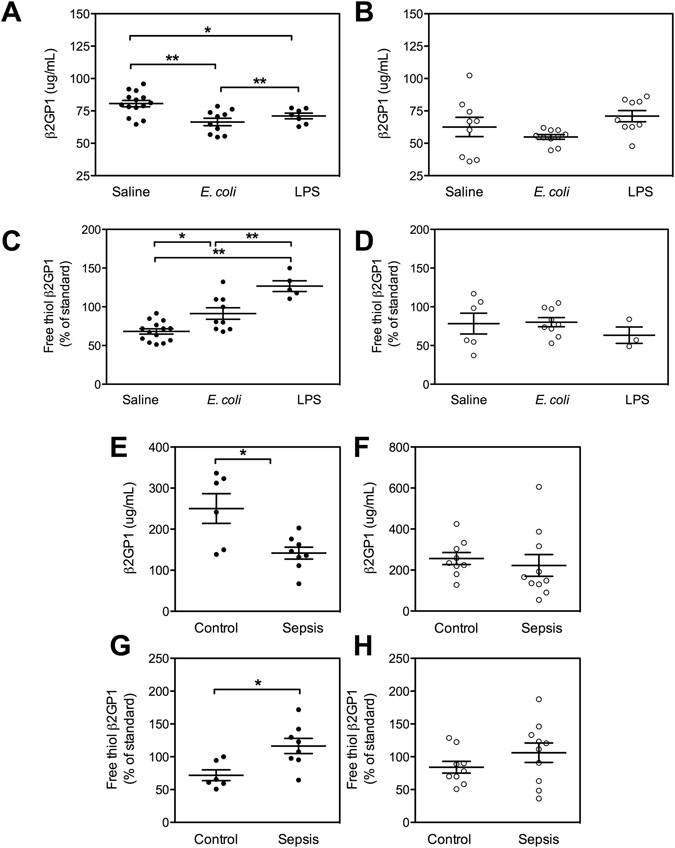
Plasma levels of total and free thiol β2GPI in mice six h post *E. coli* or LPS challenge and in human subjects with sepsis and normal controls. Total β2GPI levels (**A**). Male and (**B**). Female mice, following *E. coli* or LPS challenge (male *n* = 14 saline, *n* = 10 *E. coli, n* = 7 LPS and female *n* = 9 saline, *n* = 10, *E. coli*, *n* = 7 LPS). Free thiol β2GPI (**C**). Male and (**D**). Female mice, following *E. coli* or LPS challenge (male *n = *14 saline, *n = *10 *E. coli, n = *10 LPS and female *n = *9 saline, *n = *9 *E. coli*, *n = *10 LPS). Total β2GPI in patients with sepsis (**E**). Male and (**F**). Female patients compared to healthy controls (*n = *9 male sepsis, *n = *6 male control, *n = 8* female control, *n = *10 female sepsis). Free thiol β2GPI in patients with sepsis (**G**). Male and (**H**). Female compared to healthy controls (male sepsis *n = *8, female sepsis *n = *10, male control *n = *6, female control *n = *9,). Male = ●, Female = ○, Data represented as mean ± SEM, Mann-Whitney *p < 0.05, **p < 0.01.

Following an *E. coli* challenge total β2GPI levels decrease in male mice, whereas there was no difference in female mice (male WT saline 80.66 ± 2.46 μg/mL, *n = *10 *vs* male WT *E. coli* 66.45 ± 2.85 μg/mL*, n = *14, mean ± SEM, p < 0.003 (Fig. 4A); female WT saline 67.07 ± 8.0 μg/mL *vs* female WT *E. coli* 54.86 ± 1.83 μg/mL, mean ± SEM, *n = *10, *p = *0.278) (Fig. 4B). This *E. coli* mediated drop in total β2GPI levels is a similar response seen in previous studies which have only looked at male human subjects^3^ and male mice injected intravenously with LPS^4^.

Following an *E. coli* challenge the percentage of free thiol β2GPI in male WT mice treated with saline was 68.21 ± 3.4% of pooled normal standard, mean ± SEM, *n = *14 and for *E. coli* treated mice increased to 91.31 ± 7.45% of pooled normal standard, mean ± SEM, *n = *9, *p < *0.03, (Fig. 4C). The percentage of free thiol β2GPI in LPS treated male mice increased to 106 ± 7.73% of pooled normal standard (mean ± SEM*, n = *10, *p < *0.002) (Fig. 4C). In contrast, we observed no change in the percentage of plasma free thiol β2GPI in female mice post *E. coli* (*n = *10, *p = *0.91) or LPS challenge compared to female mice given saline (*n = *9, *p = *0.93) (Fig. 4D).

We quantified total and free thiol β2GPI levels in both male and female patients that fulfilled the criteria of sepsis^14^ and compared them to normal controls. Male patients with sepsis had a significant decrease in the plasma levels of total β2GPI (141.7 μg/ml ± 14.63 μg/ml, mean ± SEM, *n = *8) compared to normal male controls (250.3 μg/ml ± 36.19 μg/ml, mean ± SEM, *n = *6, *p = *0.03) (Fig. 4E). In contrast, there was no difference in the levels of total β2GPI in female patients with sepsis compared to female controls (Fig. 4F). The levels of free thiol β2GPI increased in male patients compared to controls (Fig. 4G). Whereas there was no difference in the levels of free thiol β2GPI in the female cohort (Fig. 4H).

### Levels of β2GPI in brain and spleen

The hypothalamus of the brain and spleen were homogenized and protein extracts were assayed for total β2GPI. There was no difference in total β2GPI between male mice injected with saline compared to male mice injected with *E. Coli* (hypothalamus 1.68 ng ± 0.84ng β2GPI/100 μg of protein (mean ± SD, n = 4) compared to 1.93 ng ± 0.53 ng β2GPI/100 μg of protein (mean ± SD, n = 4); spleen 4.15 ng ± 0.57 ng β2GPI/100 μg of protein (mean ± SD, n = 4) compared to 5.38 ng ± 2.00 ng β2GPI/100 μg of protein (mean ± SD, n = 4) respectively). For female mice injected with saline or *E. coli* there was also no difference in total β2GPI (hypothalamus 1.31 ng ± 0.44 ng β2GPI/100 μg of protein (mean ± SD, n = 4) compared to 1.48 ng ± 0.15 ng β2GPI/100 μg of protein (mean ± SD, n = 4); spleen 4.89 ng ± 1.3 ng β2GPI/100 μg of protein (mean ± SD, n = 4) compared to 4.07 ng ± 1.01 ng β2GPI/100 μg of protein (mean ± SD, n = 4) respectively).

### β2GPI influences cytokine signatures following an E. coli intravenous challenge

**Figure 5:**
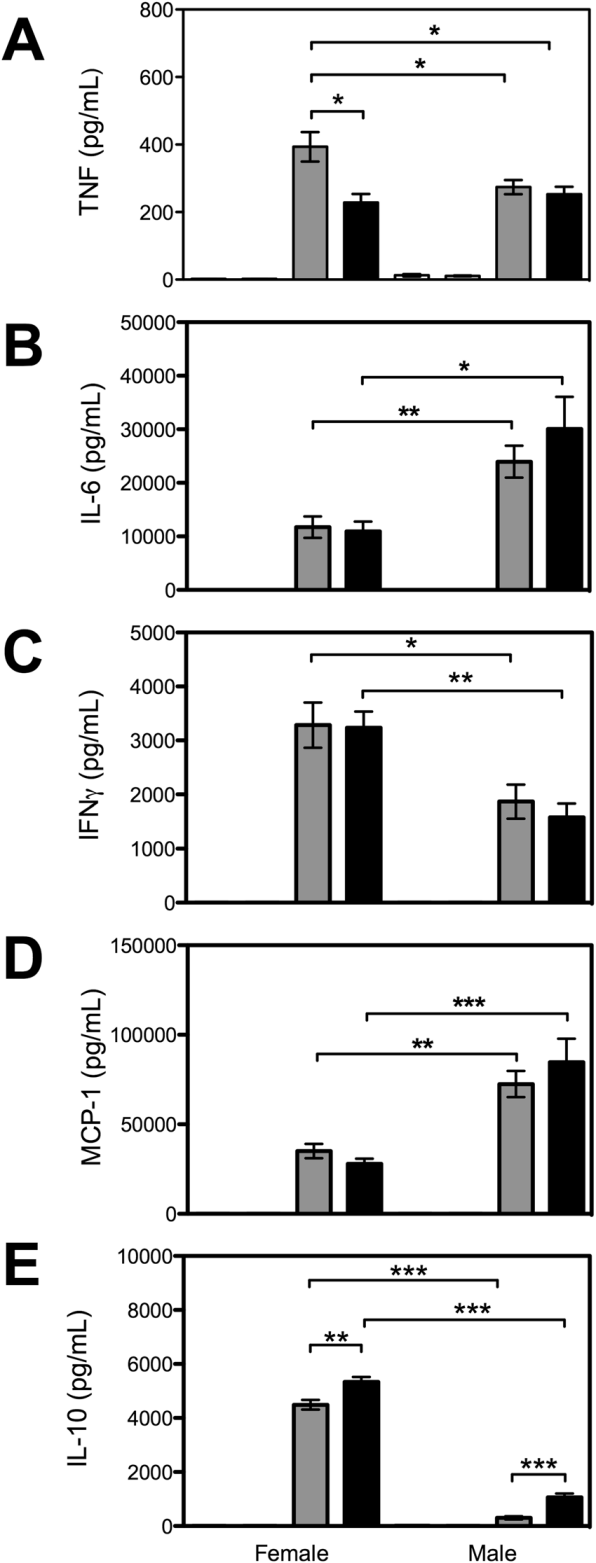
Plasma cytokine levels in mice six h following *E. coli* challenge. Saline WT = white bar; Saline β2GPI^−/−^ = light grey bar; *E. coli* WT = dark grey bar; *E. coli* β2GPI^−/−^ = black bar. Female WT *n = *8 saline and *n = *10 *E. coli;* Female β2GPI^−/−^ *n = *7 saline and *n = *10 *E. coli;* Male WT *n = *5 saline and *n = *10 *E. coli*, Male β2GPI^−/−^ *n = *5 saline and *n = *10 *E. coli*. (**A**). TNF (**B**). IL-6 (**C**). IFNγ (**D**). MCP-1 (**E**). IL-10.

Six h post *E. coli* challenge, levels of TNF, IL-6, IFNγ,MCP-1 and IL-10 were significantly increased in both male and female WT and β2GPI^−/−^ mice compared to sex matched saline treated controls (Fig. 5A–E). Following an *E. coli* challenge female WT mice produced higher levels of TNF than female β2GPI^−/−^ mice (*p < *0.02). We found no difference in plasma levels of TNF between male and female β2GPI^−/−^ mice challenged with *E. coli* (*p = *0.58) (Fig. 5A).

Following an *E. coli* challenge there was no difference in IL-6, IFNγ, and MCP-1 between sex-matched WT and β2GPI^−/−^ mice. Male WT and β2GPI^−/−^ compared to female WT and β2GPI^−/−^ mice produced higher levels of IL-6 and MCP-1 respectively (Fig. 5B,D). Female WT and β2GPI^−/−^ mice produced higher levels of IFNγ and IL-10 than male WT and β2GPI^−/−^ mice respectively (Fig. 5C,E).

### β2GPI influences cytokine production following an LPS challenge

**Figure 6:**
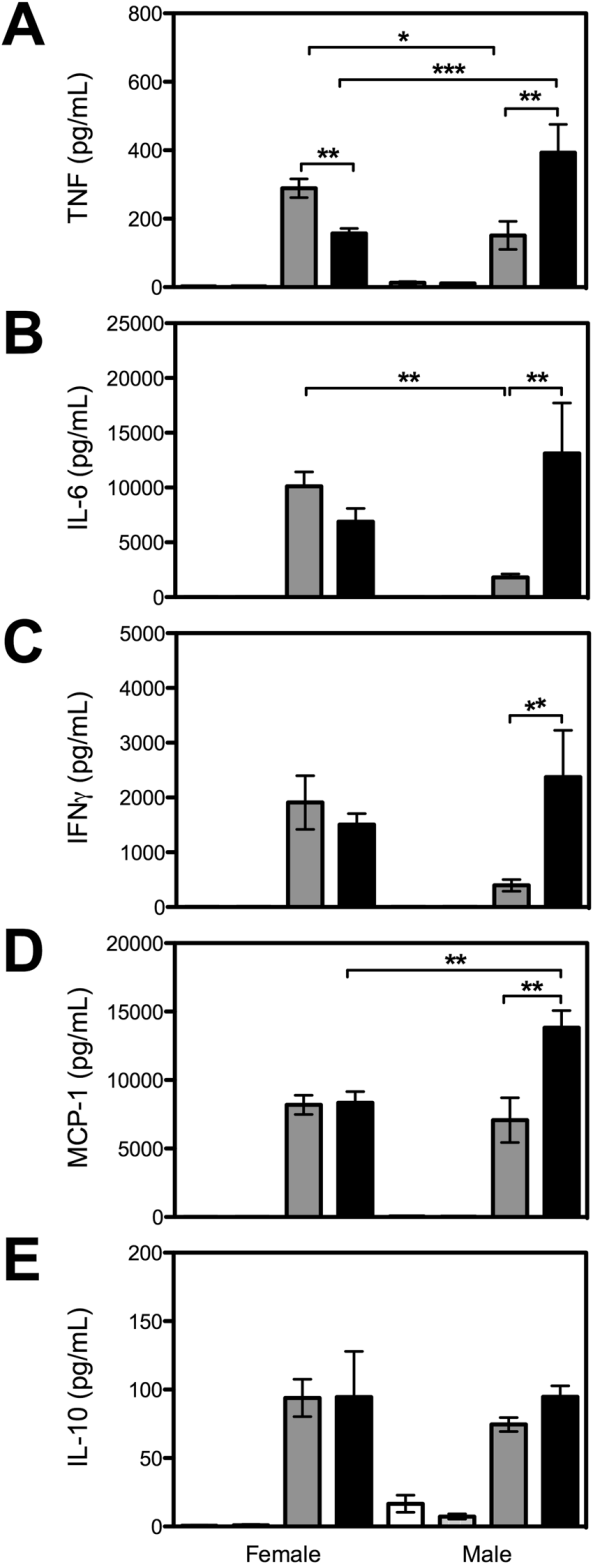
Plasma cytokine levels in mice six h following LPS challenge. Saline WT = white bar; Saline β2GPI^−/−^ = light grey bar; LPS WT = dark grey bar; LPS β2GPI^−/−^ = black bar. Female WT *n = *8 saline and *n = *8 LPS; Female β2GPI^−/−^ *n = *7 saline and *n = *8 LPS; Male *n = *5 per group. (**A**). TNF (**B**). IL-6 (**C**). IFNγ (**D**). MCP-1 (**E**). IL-10.

In response to an LPS challenge, both male and female WT and β2GPI^−/−^ mice produced higher levels of TNF, IL-6, IFNγ, MCP-1 and IL-10 compared to saline treated controls (Fig. 6A–E). Six h following an LPS challenge, female WT mice produced higher levels of TNF than female β2GPI^−/−^ mice (Fig. 6A). Whereas the male β2GPI^−/−^ had higher levels of TNF compared to WT (Fig. 6A). There was no statistical difference in the level of IL-6 between female WT and female β2GPI^−/−^ (Fig. 6B). However, the male β2GPI^−/−^ mice produced higher levels of IL-6 than the male WT mice (Fig. 6B). Male β2GPI^−/−^ mice produced higher levels of IFNγ and MCP-1 compared to the male WT (Fig. 6C,D).

### Male β2GPI deficient mice have distinct cellular profiles in the blood and spleen following an E. coli challenge

**Figure 7:**
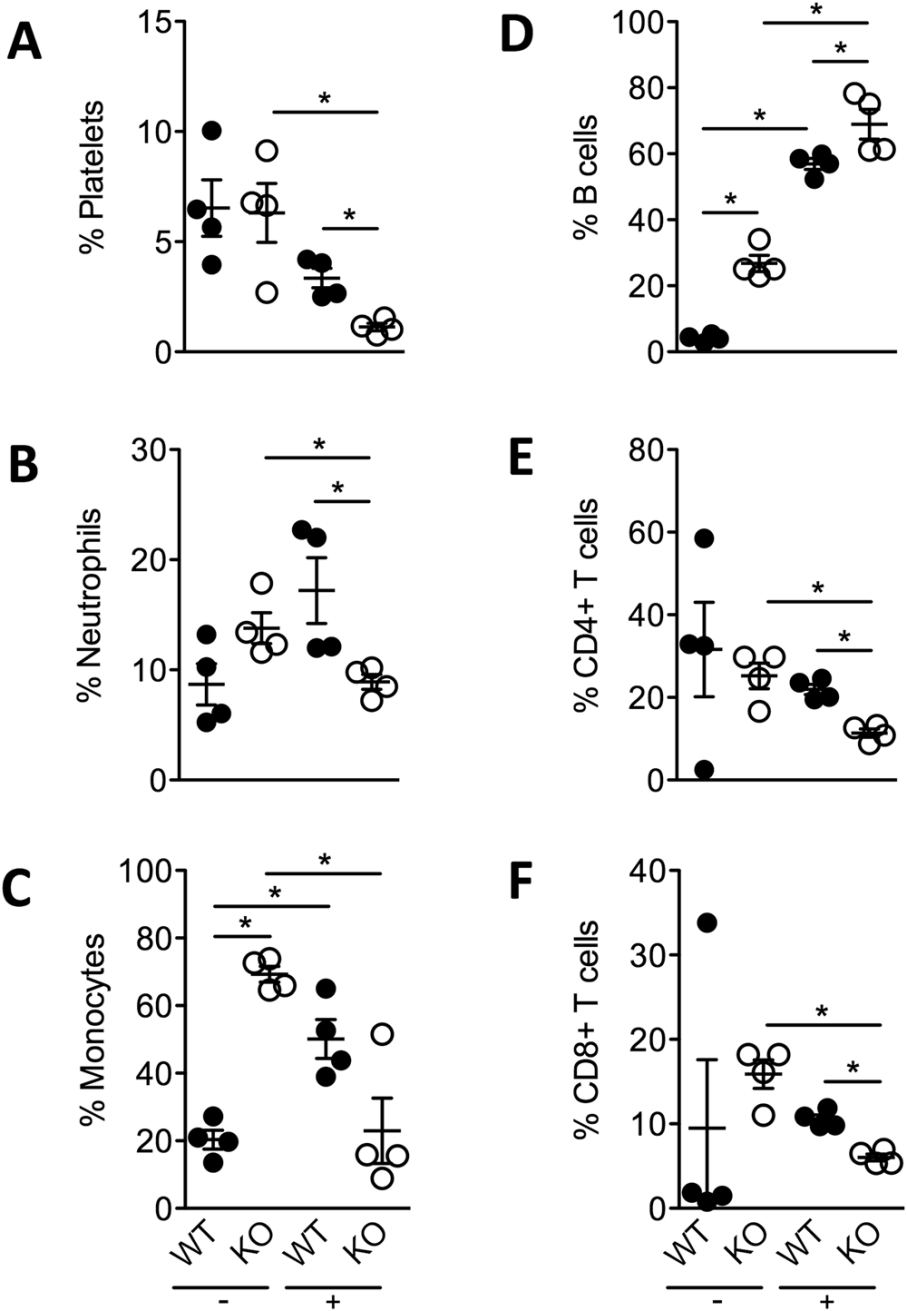
Male β2GPI deficient mice have distinct cellular profiles in the blood and spleen following an *E. coli* challenge (**A**). Percentage of circulating platelets (CD41^+^) in peripheral blood, (**B**,**C)**. Percentage of neutrophils (LY-6G^+^ and LY-6C^+^) and monocytes (CD11b^+high^) in CD45^+^ leucocytes in peripheral blood, (**D**–**F**). B cells (CD19^+^), CD4 + (CD3^+^CD4^+^CD8^−^) and CD8 + (CD3^+^CD4^−^CD8^+^) T cells in CD45^+^ splenocytes. *n = *4 per group, Male WT = ●, Male β2GPI^−/−^ (KO) = ○, “-” = saline, “ + ” = *E. coli*. Data represented as mean +/− SEM, Mann-Whitney *p < 0.05.

Since male β2GPI^−/−^ mice developed an earlier onset of severe sepsis, we characterized the cellular composition of the blood and spleen in male WT and β2GPI^−/−^ mice. Following an *E. coli* challenge, the peripheral blood platelet counts in both WT and β2GPI^−/−^ male mice decreased. However, the drop in platelets in β2GPI^−/−^ mice was significantly greater compared to the WT mice (Fig. 7A).

In the circulation, neutrophil counts increased in WT mice and decreased in β2GPI^−/−^ mice challenged with *E. coli* (Fig. 7B). Monocyte numbers in the peripheral blood were significantly higher in saline treated β2GPI^−/−^ mice compared to saline treated WT mice. However, following an *E. coli* challenge monocyte numbers increased in WT mice, yet decreased in β2GPI^−/−^ mice (Fig. 7C).

Following an *E. coli* challenge, the numbers of B cells in the spleen of β2GPI^−/−^ mice increased and both CD4 + and CD8 + T cells decreased compared to WT mice (Fig. 7D–F).

## Discussion

This is the first demonstration that the plasma protein β2GPI influences gram-negative septicaemia in a sex-specific manner following intravenous administration of live *E. Coli*. We present a role for β2GPI in controlling *E. coli* infection in the blood in both male and female mice and most strikingly we report on the differential influence of β2GPI on male and female septicaemia severity. Male mice showed a significant reliance on β2GPI for protection against the development of severe clinical septicaemia. However, β2GPI did not influence the severity of septicaemia induced by intravenous administration of *E. coli* in female mice over 24 h. We found that plasma levels of total β2GPI decreased following an *E. coli* or LPS challenge in male mice. In contrast, this phenomenon was not observed in female mice. These results suggest that the drop in total β2GPI levels in males is due to β2GPI being functionally utilized as part of the immune system’s protective response against *E. coli*. We have considered the possibility that β2GPI is redistributed from the plasma to other organs such as the brain and spleen as demonstrated by Agostinis *et al*. using fluorescent labelled human oxidized β2GPI injected in a LPS murine inflammation model^15^. Our data demonstrates that endogenous β2GPI levels do not increase in the hypothalamus or the spleen of mice injected intravenously with *E. coli*.

It has previously been shown that the murine model is relevant to the understanding of β2GPI pathobiology in infection due to the identical trends in serum and plasma total β2GPI levels in male mice^4^ and male patients^3^. The decrease in total β2GPI in the plasma, following intravenous challenge of LPS in male mice mirrored similar observations in male human subjects challenged with LPS^3^. A mean reduction of 25% in baseline levels of total β2GPI was observed in all male human subjects immediately after an LPS challenge. Patients with severe gram-negative septicemia in ICU had lower levels of β2GPI than non-septic patients^3^. We found a similar drop in total β2GPI levels in male septic patients in this study but we found no difference in the levels of total β2GPI between female septic patients and female non-septic controls. We also demonstrate that male mice challenged with *E. coli* had lower levels of plasma β2GPI compared to non-infected male mice challenged with pyrogen-free saline, whereas there was no change in total β2GPI levels in female mice challenged with *E. coli* or LPS compared to pyrogen free saline. To our knowledge this is the first report of a potential sex-specific plasma biomarker for gram negative septicaemia.

Sex differences in immune responses to various infections have previously been reported between males and females in humans and in mice as reviewed by Klein and Flanagan^13^. Females are reported to have a more efficient innate immune response than males in some studies^16^. Female mice have been reported to clear *Streptococcus pneumonia* more efficiently than male mice^17^. In our study, female WT mice had lower numbers of *E. coli* CFU compared to male WT 6 h post-administration of an identical dose of *E. coli* inoculum.

β2GPI has previously been shown to bind blood borne *E. coli* with high efficiency in a molecular based assay using whole blood^18^. We have found that WT mice have less *E. coli* CFUs in their blood than β2GPI deficient mice in both males and females. A mechanistic explanation for this is provided by the study of Nilsson *et al*., which found that anti-bacterial peptides derived from Domain V of β2GPI were generated by activated neutrophil release of granule proteases^19^. They demonstrated that the lysine rich peptides from β2GPI exhibited bactericidal activity against *E. coli* in a dose dependent manner. A major fraction of the peptide was inserted into the lipid membrane of the *E. coli* leading to extensive membrane disruption and the extravasation of cytosolic content. In the setting of *E. coli* infection neutrophils are activated and release granule proteases which we propose cleaves β2GPI *in vivo* to generate anti-bacterial peptides which we postulate is responsible for the lower *E. coli* CFU in the blood of WT compared to the β2GPI deficient mice.

LPS is negatively charged and covers over 90% of the cell wall of gram-negative bacteria. β2GPI may opsonize LPS on the *E. coli* surface. The interaction of LPS with β2GPI may induce a conformational change in β2GPI, from closed to open^3^. This conformational change may allow binding to lipoprotein receptor-related protein (LRP) family-receptors rather than Toll like receptor 4 (TLR4), enabling internalization by macrophages or monocytes. This may lead to more efficient bacterial clearance and reduced release of pro-inflammatory cytokines through reduced activation of TLR4 and this may be another mechanism to account for the lower number of *E. coli* CFUs in the WT mice compared to the β2GPI deficient mice.

We found an increase in the plasma of free thiol β2GPI generated in WT male mice in response to *E. coli* but not in female WT mice. This sex-specific correlation in the levels of free thiol β2GPI was also seen in male and female patients with sepsis. Free thiol β2GPI can be generated when oxidized β2GPI is treated with an oxidoreductase such as thioredoxin-1^9^ and this conversion is upregulated in the presence of LPS^4^. *In vitro*, free thiol β2GPI protects endothelial and retinal epithelial cells from hydrogen peroxide induced cell injury^10, 11^. In the context of septicaemia, we speculate that the generation of free thiol β2GPI may be protective of the host’s vascular endothelium. Another possibility is that the free thiols in Cys-288 and Cys-326 of β2GPI may interfere with the signaling of the MD2-TLR4 receptor complex as we have previously proposed^4^. It is relevant to note that Cys-288 is in the central part of the most potent anti-bacterial peptide region from Domain V of β2GPI^19^. Domain V free thiols may exert an antibacterial effect by interfering with disulphide bond formation in the bacterial cell wall, which are critical for organism viability^20^. Domain V peptides with free thiols may be developed to be used as novel antibacterial agents.

We propose that in males, β2GPI serves a critical and active role in protecting against *E. coli* septicemia; whilst in females the role of β2GPI appears to be redundant. What protective factors are at play in females to account for why β2GPI does not have as an important role in female innate immunity has not been determined in this study. Females are more susceptible to urinary tract infections with *E. coli* ^21^ because of their anatomy and reproductive-gestation capacity. We speculate this may account for added evolutionary pressure on females compared to males to develop additional innate immunological mechanisms, up and above β2GPI, to efficiently clear *E. coli* from the systemic circulation and to limit the dysregulated immune response associated with such an infection. If these redundant immunological mechanisms did not evolve in females compared to males the consequences for increased susceptibility to *E. coli* infection during pregnancy would presumably affect their reproductive capacity to deliver healthy offspring.

Overall, our findings support a role for β2GP1 protecting male mice from gram-negative septicaemia and modulating immune responses to *E. coli*.

Our clinical data suggest β2GPI may serve a similar role in humans. The current study focuses on the role of β2GPI in modulating gram negative septicaemia rather than sepsis. Sepsis is a complex dysregulated immune response leading to multiorgan failure which requires distinct mouse models such as the caecal puncture peritoneal infection model, which is outside the scope of this current study.

Our study delineates the role of β2GPI in murine septicaemia though not in murine sepsis, however our preliminary patient data suggest that examining the role of β2GPI in sepsis models in the future is a reasonable avenue to pursue.

## Methods

### Reagents

The following reagents were used in this study: wild-type smooth strain *Escherichia coli* (*E. coli*) (O55:B5, ATCC® 12014™), LPS from *E. coli* (0111:B4) and RIPA buffer with protease inhibitor cocktail were from Sigma-Aldrich, St Louis, Missouri), pyrogen-free saline (Thermo Fisher Scientific, North Ryde, New South Wales, Australia), Cytometric Bead Array (CBA) Mouse Inflammation Kit (BD Biosciences, North Ryde, New South Wales, Australia), bovine serum albumin (BSA) (Sigma-Aldrich), alkaline phosphatase (AP) conjugated anti-mouse (Sigma-Aldrich), anti-rabbit and anti-human IgG (Sigma-Aldrich), β2GPI (Hematologic Technologies, Essex Junction, Vermont), isotype control rabbit polyclonal IgG (BD Biosciences) and red blood cell lysis buffer (eBioscience, San Diego, California). Triple-Pure High Impact Zirconium 1.5 mm beads (Benchmark Scientific, NJ) Affinity purified murine IgG2, anti-β2GPI monoclonal antibody and affinity purified polyclonal rabbit anti-β2GPI antibody were produced as previously described^22, 23^.

PE-rat anti-mouse CD41, APC-Cy7 rat anti-mouse CD45, BV510 hamster anti-mouse CD3e, PerCP-Cy5.5 rat anti-mouse CD4, PE-Cy7 rat anti-mouse CD8a, APC rat anti-mouse CD19, V450 rat anti-mouse LY-6G and LY-6C, PE rat anti-mouse CD11b and mouse Fc block^TM^ were purchased from BD Biosciences and used at 0.2 mg/mL.

### Ethics Statement

The University of New South Wales Australia Animal Care and Ethics Committee (ACEC) approved all experimental procedures and the animals were housed under pathogen-free conditions (Approval #: 15/71B). All protocols adhered with the *Animal Research Act 1985* and the *Australian Code for the Care and Use of Animals for Scientific Purposes 8th Edition* (2013).

The South Eastern Sydney Local Health District Human Research Ethics Committee (HREC) (HREC 15/252, LNR/15/POW/461) approved the clinical study. Informed consent was received from all patients and healthy controls prior to sampling and all protocols adhered to the *National Health and Medical Research Council’s (NHMRC) National Statement on Ethical Conduct in Human Research (2007)*, *NHMRC and Universities Australia Australian Code for the Responsible Conduct of Research (2007) and the CPMP/ICH Note for Guidance on Good Clinical Practice*.

### Animals

Male and female, age matched mice (8–10 weeks old) were used in this study. C57BL/6 WT mice were purchased from the Animal Resources Centre (Perth, Australia) and C57BL/6 β2GPI^−/−^ mice were generated as previously described^24^.

### Escherichia coli

*E. coli* were grown on horse blood agar (HBA) (Oxoid, Adelaide, South Australia, Australia) plates for 18 h in oxygen at 37**°**C. *E. coli* suspensions were made up in sterile PBS. The final CFU concentrations were determined using a specific OD_600_ value using Tecan Infinite® 200 PRO spectrophotometer and adjusted accordingly.

### Models of inflammation and gram negative septicaemia

Mice received either a retro-orbital intravenous injection of 1 μg/gram body weight (gbw) of LPS or 10^8^ colony-forming units (CFU) of *E. coli* or an equivalent volume of pyrogen-free saline as a vehicle control. Final LPS concentrations were adjusted using pyrogen-free saline. *E. coli* were washed twice with pyrogen free saline, resuspended in 200 μL pyrogen-free saline and injected intravenously.

Each mouse was monitored every hour for six h, the timing of scoring evaluated for clinical signs of septicaemia and allocated a clinical score based on parameters adapted from Huet *et al*., and Shrum *et al*.^25, 26^ (Table 1).

### Plasma preparation

For mice injected with LPS or *E. coli* at 6 h post-injection, or *E. coli* at 24 h post-injection blood was collected via retro-orbital venepuncture under anaesthesia in 0.129 mol/L sodium citrate (ratio of blood to anticoagulant 4:1). Samples were further processed to achieve platelet free plasma as published previously^27^ and stored at −80**°**C until they were analysed.

### Bacterial load determination

At 6 h post-injection, samples of spleen and liver tissue from *E. coli* challenged mice were weighed and homogenized in up to 1 mL of PBS. 100 μL of citrated blood from these mice was diluted in 900 μL of PBS. Spleen and liver homogenates and blood were serially diluted 1:10 in PBS and plated onto separate HBA plates. The plates were incubated for 18 h in oxygen at 37**°**C. Colonies were counted on each plate by an observer blinded to the experimental groups and the number of CFU per mL for blood were determined for each sample. Spleen and liver counts were normalized to sample weight (g) and blood counts normalized to 100 μL blood volume.

### Cytokine measurement

The levels of proinflammatory cytokines interleukin-6 (IL-6), monocyte chemoattractant protein-1 (MCP-1), interferon gamma (IFNγ), tumour necrosis factor (TNF) and anti-inflammatory cytokine interleukin 10 (IL-10), were quantified in the plasma samples using BD Cytometric Bead Array (CBA) Mouse Inflammation Kit and BD FACSCanto II according to the manufacturer’s instructions.

### Preparation of brain and spleen tissue extracts for quantitation of β2GP1

Female and male, WT and β2GPI-/- mice were anaesthetized and perfused with phosphate-buffered saline (PBS) through the left ventricle following cardiac puncture. After the mice were euthanized, the whole hypothalamus and spleen were surgically excised and snap frozen in liquid nitrogen. Tissues were added into a 2 ml v-bottom tube containing 100 μl of RIPA buffer (with protease inhibitor cocktail) and 10 Triple-Pure High Impact Zirconium 1.5 mm Beads (Benchmark Scientific). The tubes were added to the BeadBug 3 Microtube Homogenizer and shaken for 180 seconds to achieve homogenization. The samples were then centrifuged at 15000 rpm at 4 °C for 20 min and the supernatant was transferred to a new tube and stored at −80 °C. The concentration for total protein of each sample was measured using the Micro BCA kit (Pierce).

### Measurement of total and free thiol β2GP1

Plasma was assayed for the levels of total and free thiol β2GPI using ELISAs as published previously^28^.

### Flow cytometry

Fluorochrome-conjugated monoclonal antibodies directed against selected cell surface antigens were used to identify platelets, neutrophils, monocytes in mouse peripheral blood and B cells and CD4 + and CD8 + T cells in the spleen. Spleens were isolated at the time of euthanasia and processed to single cell suspensions in PBS. Whole blood and unlysed splenocytes were stained for CD41. Following red blood cell lysis, cells were washed with PBS and blocked using Mouse Fc block^TM^ for 5 min at 4**°**C. Cells were stained with antibodies for 15 min at RT in the dark, washed and resuspended in 100 μL PBS prior to analysis. Fluorescence minus one and positive controls were used to assist with gating. Samples were acquired on BD FACSCanto II (BD Biosciences) on low speed and data was analysed using Kaluza Analysis Software version 1.5 (Beckman Coulter, Lane Cove West, NSW, Australia). Cells were gated for leucocytes according to FCS vs SSC intensity and on FSC-area vs FSC-height to discriminate singlet cells from doublet cells. Singlet cells were then plotted on a dot-plot for CD45 before gating further for sub-populations.

### Clinical study

Plasma was collected from fifteen adult volunteers without sepsis (male = 6, female = 9) and eighteen patients with sepsis (male = 8, female = 10). Consent was received from all patients and controls prior to sampling. Patients were deemed septic if they fulfilled the international criteria for sepsis^14^. Control samples were collected from adult volunteers with no history of thrombosis, malignancy or active infections.

### Statistical analyses

Statistical significance was determined using GraphPad software (version 5 for Mac OS). Data was log transformed for representation of CFU. Differences in plasma levels of total and free thiol β2GPI, cytokine levels, CFU and cellular compositions were determined using the non-parametric Mann Whitney Test. Comparison of severity score was performed using the Gehan-Breslow-Wilcoxon Test (Chi square). *p < 0.05 were deemed significant.

### Data availability

All data generated or analysed during this study are included in this published article.
